# Learning and prospective recall of noisy spike pattern episodes

**DOI:** 10.3389/fncom.2013.00080

**Published:** 2013-06-21

**Authors:** Karl Dockendorf, Narayan Srinivasa

**Affiliations:** Information and System Sciences Lab, Center for Neural and Emergent Systems, HRL Laboratories LLCMalibu, CA, USA

**Keywords:** spiking, STDP, learning, sequences, prospection, preplay, replay, memory

## Abstract

Spike patterns *in vivo* are often incomplete or corrupted with noise that makes inputs to neuronal networks appear to vary although they may, in fact, be samples of a single underlying pattern or repeated presentation. Here we present a recurrent spiking neural network (SNN) model that learns noisy pattern sequences through the use of homeostasis and spike-timing dependent plasticity (STDP). We find that the changes in the synaptic weight vector during learning of patterns of random ensembles are approximately orthogonal in a reduced dimension space when the patterns are constructed to minimize overlap in representations. Using this model, representations of sparse patterns maybe associated through co-activated firing and integrated into ensemble representations. While the model is tolerant to noise, prospective activity, and pattern completion differ in their ability to adapt in the presence of noise. One version of the model is able to demonstrate the recently discovered phenomena of preplay and replay reminiscent of hippocampal-like behaviors.

## Introduction

The CA3 region of the mammalian hippocampus is a typical example of a recurrent neural network *in vivo* (Hasselmo et al., 1995; Rolls, 2000; Kobayashi and Poo, 2004; Kesner, 2007; Li et al., 2010). It is known these recurrent networks are well-suited for learning pattern sequences and pattern completion (Treves and Rolls, 1992; Hopfield, 1995; Káli and Dayan, 2000; Gold and Kesner, 2005; Leutgeb et al., 2007; Yassa and Stark, 2011). While in general neural networks (Graves et al., 2009) have shown their ability to perform tasks on highly corrupted data, spiking neural network (SNN) models are often sensitive to input scale and must be carefully tuned to generate the desired output (Buonomano, 2005). These issues are amplified in recurrent SNNs where instabilities can result in cascades of activity even with slight input perturbations (Gerstner and Kistler, 2002). Furthermore, biological networks are exposed to a great degree of input variability that can cause many simulated SNNs to fail.

In order to address input variability, recent model implementations showed that it is possible to globally scale the synaptic weight update on afferent synapses so as to constrain the cumulative weight to an artificial limit. This helps to also maintain stability in the model (Song et al., 2000; Van Rossum et al., 2000). However, presynaptic spike timing is primarily maintained locally at each synapse without evidence *in vivo* for an instantaneous global rescaling of all afferent synaptic weights. One biologically plausible solution is to incorporate homeostatic regulation. Several forms of homeostasis exist in biological networks that occurs at many timescales and is critical for the stability of these networks (Malinow and Malenka, 2002; Renart et al., 2003; Turrigiano and Nelson, 2004; Deeg, 2009; Turrigiano, 2011). Moreover, modeling homeostasis allows for a self-adjustment and overall scaling of input synaptic weights to neurons in a network and can help compensate for this input variability.

Learning of pattern sequences through the use of plasticity has been studied before (Berthouze, 2000; Arthur, 2006). These methods have relied on learning associations with sequences of on-going reliable and repetitive background activity. However, repeatable sequences have only been detected during task-related activity (Itskov et al., 2011). Moreover, the sequencing of activity (Diba and Buzsaki, 2007) is likely to be based on self-sustaining recurrent pathways of activity in the network due to previous association with sensory and spatial activity.

In this paper, we present three variations of a recurrent SNN for learning spatiotemporal patterns of activity using spike-timing dependent plasticity (STDP) with homeostatic regulation of activity. This model can learn to integrate patterns into pattern sequences from multiple noisy presentations and complete previously learned patterns from partially available data. Furthermore, stable pattern learning can be achieved with synaptic weight changes despite relative differences in intensity of the input patterns. The network demonstrates both *prospective* firing of activity and pattern completion while maintaining stability without the need to balance the input weights directly. The lessons learned from the development of these models are discussed and potential opportunities for handling their limitations are noted.

## Materials and methods

### Simple network model

**Figure 1:**
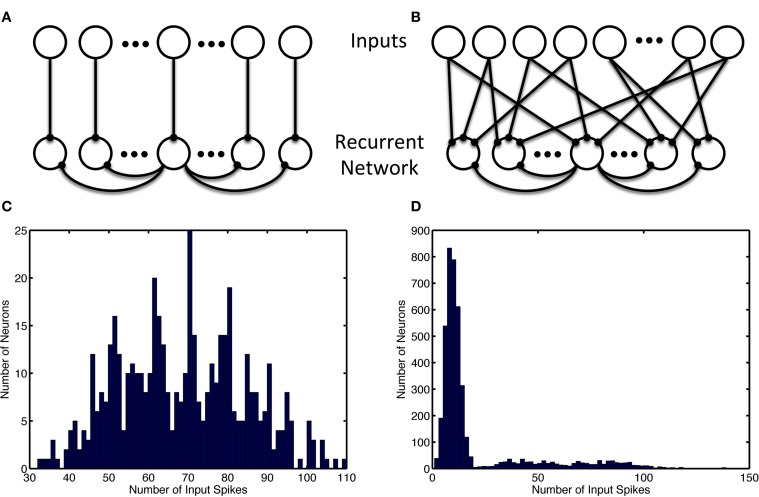
**Recurrent and input network structure and distribution**. Recurrent connections are shown only for the center recurrent neuron. **(A)** Input structure of the simple model with 1-to-1 network inputs, recurrent neurons receive input from one and only one input neuron. **(B)** Input structure of the alternate model with many-to-many network inputs, each recurrent neurons receives input from 12 random input neurons (which project 3 outputs each). **(C)** Unbalanced usage of the input space with and without input neuron allotment. Histogram shows the distribution of the number of input spikes due to a specific neuron. **(C)** Shows the activity of 480 input neurons when the patterns are chosen consist of separate groups of neurons used for simple model inputs. **(D)** Show the multimodal nature of the 8000 input neurons' activity when neurons are selected randomly without regard for usage in other ensembles, as is the case in the alternate model. The leftmost mode represents noise, while the successively rightward modes of the distribution represent redundant usage of the input neurons in the episode patterns.

The simple network model is comprised of a recurrent population of Izhikevich neurons (Izhikevich, 2007a) with the default parameters from Table 1. Each neuron connects to a fraction (*pe* and *pi*) of all other excitatory and inhibitory neurons (see Figure 1) with both excitatory and inhibitory connections having a random delay (τ*e*^delay^ and τ*i*^delay^). The network is constructed such that for each excitatory connection, an inhibitory connection is also made between those neurons. For simplicity, one neuron is used for both connections, however, in an alternate larger model with different input scheme these dual paths are implemented with separate inhibitory and excitatory neurons. The recurrent network structure itself is random but balanced such that each neuron has an equal number of efferent and afferent connections and all neurons have the same number of connections.

**Table 1:** **Simple model parameters**.

**Network attribute**	**Parameter**	**Value**
Synaptic conductances	*E*_*i*_	−81 mV
	*E*_*e*_	0 mV
	τ_*i*_	50 ms
	τ_*e*_	35 ms
	τ^delay^_*i*_	[1, 5] ms
	τ^delay^_*e*_	[11, 16] ms
	*w*^max^_*i*_	0.8 nS
	*w*^min^_*i*_	0.5 nS
	*w*^max^_*e*_	0.8 nS
	*w*^max^_*i*_	0 nS
Excitatory triplet STDP	τ^LTP^_*e*_	20 ms
	τ^LTD^_*e*_	25 ms
	δ^LTP^_*e*_	0.020 nS
	δ^LTD^_*e*_	0.024 nS
Inhibitory top hat STDP	τ^LTP^_*i*_	40 ms
	τ^LTD^_*i*_	700 ms
	δ^LTP^_*i*_	0.02 nS
	δ^LTD^_*i*_	0.05 nS
Homeostatic excitatory scaling	τ_ω_	600 s
	ω_*D*_	[0.25, 0.35] Hz
1-to-1 simple network	*N*_*N*_	480
	*p*_*i*_	1
	*p*_*e*_	1
Neuron model	*a*	0.02
	*b*	0.5
	*c*	−40 mV
	*d*	55
	*v*_*i*_	−45 mV
	*v*_*r*_	−60 mV
	*v*_*p*_	40 mV
	*C*	50 pF
	*k*	0.5
Noise	ν	0.05 Hz

Neuron dynamics (Izhikevich, 2007a) are described by three variables, membrane voltage (*v*), recovery (*u*), and excitatory scaling of afferent synaptic conductances (*s*, long-term homeostatic parameter):

(1) $$v^{\prime }=\frac{1}{C}(k(v-v_{r})(v-v_{t})-u+I_{\text{syn}}+I_{\text{noise}})$$

(2) $$u^{\prime }=a(b(v-v_{r})-u)$$

(3) $$s^{\prime }=\frac{\omega_{D}}{\tau_{\omega }}$$

where *I*_noise_ is zero mean gaussian noise with σ = 80 pA, ω_*D*_ is the target firing rate, and τ_*w*_ is the time constant. *I*_syn_ is composed of inputs from *N* excitatory and *M* inhibitory synapses:

(4) $$I_{\text{syn}}=\sum_{j=1}^{N}s\cdot g_{j}(E_{e}-v)+\sum_{k=1}^{M}g_{k}(E_{i}-v)$$

where *g*_*j*_ and *g*_*k*_ are the excitatory and inhibitory synaptic conductances respectively. Upon action potentials (*v* ≥ *vp*),

(5) $$v=c,\;u=u+d,\text{ and }s=s-\frac{1}{\tau_{\omega }}$$

Integration was performed using Euler's method for all variables [except *v* where a hybrid method (Izhikevich, 2010) was used] with a simulation time step of 0.5 ms.

The network model is designed to have inhibitory and excitatory connectivity between neurons so that the effective pairing between any group of co-active neurons (an ensemble) and another neuron scales from net inhibitory to net excitatory for a given ensemble. Therefore, a direct connection from a neuron to another neuron maybe strongly excitatory, while the indirect connectivity between the presynaptic and postsynaptic neuron maybe inhibitory depending on the currently active ensemble. A network with the aforementioned properties can then associate positively or, by default, negatively each neuron with each ensemble. As long as the coding of the inputs and relative connectivity of the network spread the representation of ensembles such that these net effects from ensembles to any neuron can be modulated, new patterns can be learned while minimally effecting the coding of other ensembles (see Results). Thus, these networks exhibit the ability to learn or recall spike patterns whether they code for sequential spatial, sensory, or other data.

Two different forms of synaptic plasticity are used for the model, one for excitatory and one for inhibitory synapses. The inhibitory synapse rule is symmetrical, and functionally implements a rule where co-active neurons reduce their inhibitory coupling, but neurons that fire independently have strong inhibitory connectivity. Inhibitory plasticity uses an inverted top-hat shaped symmetric STDP curve, which is similar to a Mexican-hat plasticity curve (Caporale and Dan, 2008; Srinivasa and Jiang, 2013). Upon presynaptic or postsynaptic action potential:

(6) $$△w_{i}=\{\begin{array}{ll} \delta_{i}^{\text{LTD}} & \text{if}\left|t_{\text{post}}-t_{\text{pre}}\right|<\tau_{i}^{\text{LTD}}\\ \delta_{i}^{\text{LTP}} & \text{else if}\left|t_{\text{post}}-t_{\text{pre}}\right|\leq \tau_{i}^{\text{LTP}}\\ 0 & \text{otherwise} \end{array}$$

where *w* is constrained to 0 ≤ *w*_*i*_ ≤ *w*^max^_*i*_, τ^LTD^_*i*_ is the time window of long-term depression (LTD) for inhibitory STDP. The parameters δ^LTD^_*i*_ and δ^LTP^_*i*_ correspond to the change in synaptic weights for inhibition during LTD and long-term potentiation (LTP), respectively. The terms *t*_pre_ and *t*_post_ correspond to the time at which presynaptic and postsynaptic spike events occur.

Complementary to the inhibitory weight changes, excitatory changes are asymmetric and strengthen synapses that contribute to the causal activation of the postsynaptic neuron and weaken those that are activated in reverse order unless the postsynaptic neuron activates again. Excitatory plasticity follows a triplet-based STDP rule (Pfister and Gerstner, 2006) and is described by the dynamics when presynaptic or postsynaptic action potentials occur:

(7) $$△w_{e}=\{\begin{array}{ll} (\delta_{e}^{\text{LTP}}+△w_{e}^{\text{LTD}})e^{\frac{t_{\text{pre}}-t_{\text{post}}}{\tau_{e}^{\text{LTP}}}} & \text{if }t_{\text{post}}-t_{\text{pre}}\geq 0\\ \delta_{e}^{\text{LTD}}e^{\frac{t_{\text{pre}}-t_{\text{post}}}{\tau_{e}^{\text{LTP}}}} & \text{if }t_{\text{post}}-t_{\text{pre}}\;<\;0\; \end{array}$$

where *w* is constrained to 0 = *w*_*i*_ = *w*^max^_*i*_ and Δ*w*^LTD^_*e*_ is the change due to the last depression event. The parameters δ^LTD^_*e*_ and δ^LTP^_*e*_ correspond to the change in synaptic weights for excitation during LTD and LTP, respectively and τ^LTP^_*e*_ is the time window of LTP for excitatory STDP. Excitatory synaptic weights are initialized with values from a uniform distribution on the interval [0, 0.1 *w*^max^_*e*_).

### Alternate network model

An alternate model of 2000 excitatory and 500 inhibitory neurons was used for some simulations with several other modifications detailed below. Excitatory neurons were recurrently connected with other excitatory neurons with probability *pee* and with the inhibitory neurons with probability *pei*. Inhibitory neurons were recurrently connected with other inhibitory neurons with probability *pii* and with the excitatory neurons with probability *pie*, see Table 2.

**Table 2:** **Alternate model parameter modifications**.

**Network attribute**	**Parameter**	**Value**
Network structure	*N*^*e*^_*N*_	2000
	*N*^*i*^_*N*_	500
	*p*_*ii*_	0.3
	*p*_*ie*_	0.3
	*p*_*ei*_	0.3
	*p*_*ee*_	0.3
Neuron model	*C*	500 pF
	*E*_*e*_	30 mV

The alternate model used Izhikevich's fast spiking (FS) model for inhibitory neurons and increased the membrane capacitance, *C*, of the neuron model to 500 pF for the excitatory neurons. Furthermore, the excitatory reversal potential, *Ee* was increased to 40 mV to prevent excitation lock that could occur with the increased membrane capacitance. Excitatory-excitatory and excitatory-inhibitory connections used the aforementioned triplet STDP rule, while inhibitory-excitatory connections used the inverted top-hat STDP rule and inhibitory-inhibitory connections used a non-inverted top-hat STDP rule. These inhibitory connections were also changed to an order of magnitude faster conductance decay time constant. A single inhibitory feedback neuron with long decay time constant was added with connections to and from all excitatory neurons; these connections were non-plastic.

### Preplay model

Patterns of place cell activity are preplayed in forward order or replayed in reverse order at the beginning and end, respectively, of a linear track (De Almeida et al., 2007). Similarly, one version of our model demonstrates preplay and replay of activity. Although less biologically plausible, this model used the same input and recurrent connection scheme and was the same size as the simple model with a few exceptions. Firstly, each neuron was the regularly spiking simple four parameter model (Izhikevich, 2003) but with membrane capacitance 1000× greater, the *a* parameter 1000× smaller, and the *d* parameter 10× smaller. Secondly, weight changes were larger with an inverted window for inhibitory STDP (this was allowable since the network was more stable due to higher capacitances and slower postspike recovery). The parameter settings for this model are summarized in Table 3.

**Table 3:** **Preplay model alterations**.

**Network attribute**	**Parameter symbol**	**Value**
Time constant for recurrent inhibitory synaptic conductances	τ_*i*_	65 ms
Time constant for recurrent excitatory synaptic conductances	τ_*e*_	120 ms
Time window for LTP portion of the inhibitory STDP function	τ^LTP^_*i*_	140 ms
Time window for LTD portion of the inhibitory STDP function	τ^LTD^_*i*_	56 ms
Excitatory reversal potential of neuron	*E*_*e*_	30 mV

### Network input

**Figure 2:**
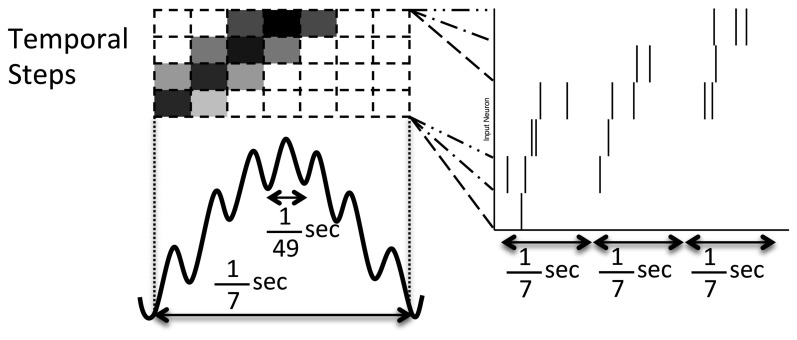
**Pattern of noisy but structured network inputs**. Every 1/7 of a second (low frequency), part of a spike episode is input to the network. Each episode is split into 30 temporal steps, each associated with a few input neurons. Groups of four temporal steps are presented sequentially in time (in successive high frequency, 1/49 s, blocks) with the current step being presented at the middle of the low frequency cycle (where the darkness of the high frequency blocks drawn above relate to the increased probability of a spike). In each successive low frequency block, the current temporal step is advanced by one, similar to activity reported in Maurer and McNaughton (2007).

Two network input structures were considered: a 1-to-1 input neuron to recurrent neuron model and a many-to-many input model where input neurons spread output onto three recurrent neurons and each recurrent neuron receive 12 connections from input neurons. 1-to-1 network inputs are used with the simple model and preplay model; the many-to-many network input structure is used with the alternate model. In the simulations with 1-to-1 inputs, each neuron receives simulated spiking input through a single large, non-plastic, excitatory synapse from an input neuron so that a spatiotemporal episode can be strongly forced onto the downstream recurrent network model. This strong influence results in the recurrent network having a high likelihood to spike in a similar manner as the upstream inputs. This input drive is modeled as noisy frequency modulation of input spikes as shown in Figure 2.

Twelve episodes were constructed each consisting of 25% of the network and segmented into 30 temporal steps. These episodes were presented in blocks of four episodes each and consisting of 100% of the network for the 1-to-1 network input structure. In each block, the episodes were presented at random (from a uniform distribution) and with overlap of four temporal steps. The current step is presented with the last few patterns in decreasing intensity each low frequency period. Neurons comprising each temporal step are activated simultaneously (with one high frequency period of jitter) for each active spatiotemporal step. As each episode is presented, one neuron out of each temporal step is selected at random to be removed from that presentation of the episode.

In the alternate model, the episodes are encoded in the same manner. However, the input neuron allotment to each episode was performed without regard for overlap and redundant usage during each group of episodes (see Figure 1). Additionally, a larger network was used and as a result each temporal step consisted of 14 neurons as opposed to 4.

In the preplay model, during each episode presentation each temporal step was repeated 7 times before proceeding to the next temporal step, whereas in the simple and alternate models each step occurred once per episode.

For all models, noise was injected into the network by adding spontaneous action potentials from the input neurons at a rate of ν = 0.05 Hz per neuron in addition to the somatic gaussian current input. A single, fast inhibitory, input neuron projects to the entire recurrent network with a fixed, but varied time delay (1–4 ms). The inhibitory input neuron supplies the network with high frequency modulated action potentials at a rate of 250 Hz.

### Simulation and analysis

Simulations were performed using a custom C/C++ MPI-based simulator and run 2–9× real time on a 2 GHz quad-core i7 MacBook Pro with 8 GB of RAM. Spike data and weights were analyzed using MATLAB. Local linear embedding (LLE) of weights was performed using the MATLAB Toolbox for Dimensionality Reduction freely available at http://homepage.tudelft.nl/19j49/Matlab_Toolbox_for_Dimensionality_Reduction.html

## Results

The results are summarized for the models as follows: the simple model with the complete usage and non-overlapping allotment of input neurons, the alternate model with the random allotment of inputs, and the preplay model with varied input types. In most cases, inputs are partially presented, noisy, and probabilistic (in their occurrence) resulting in a disproportionate use of input neurons (Figures 1, 2). It was necessary to use the alternate input mapping when using random allotment to prevent the growth of homeostatic scaling and bursting (and recruitment of the entire network) in the recurrently connected neurons not assigned to an ensemble (data not shown). This connection strategy ensures that most recurrent network neurons will receive some input, since the fraction of neurons without active inputs is small. Additionally, a many-to-many input architecture amplifies the amount of noise by increasing the number of input noise sources to each neuron in the recurrent network, which prevent the uncontrolled growth of the homeostatic parameter through direct activation of the neuron and indirectly by promoting random changes in its weights that may result in the “unused” neuron becoming active in a random ensemble without direct stimulation.

### Learning episodes

**Figure 3:**
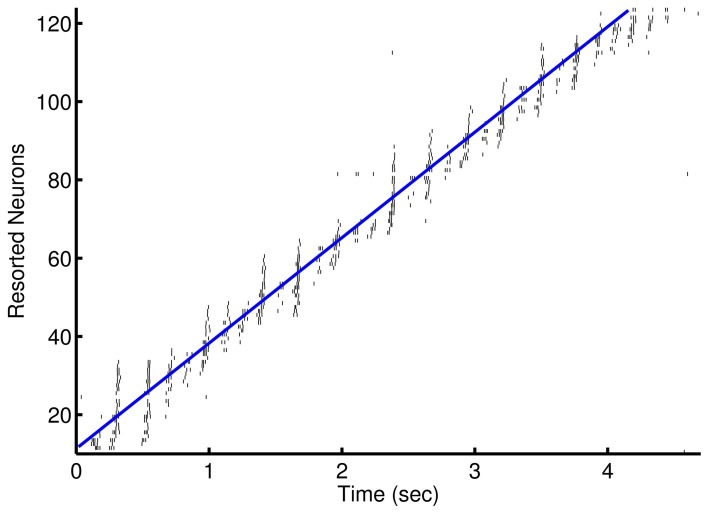
**Prospective spiking activity in the simple model**. Example raster plot of activity in the recurrent network after training of an episode in the 1-to-1 network. Neuron have been resorted by the mean time of activity in the interval. Spikes near and to the left of the blue line are, in general, prospective activity.

The use of low and high frequency modulated inputs force short, high frequency bursts of spikes that replicate a sequential long time scale procession in temporal steps. After learning, the sequential inputs activate subsequent nodes prospectively (see Figure 3). The net inhibitory effect of on-going firing limits prospective firing to a handful of future temporal steps. The network demonstrates a cascade of activity during each low frequency cycle. This cascade is preempted by the intermittent high frequency inhibition and terminated by an increase in low frequency inhibition and due to refractoriness of the neurons. The beginning of the next low frequency cycle is marked by the input stimulus that excites another cascade of activity.

**Figure 4:**
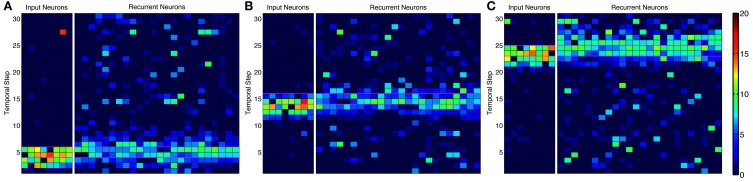
**Activity in alternative network model**. Instantaneous firing rate of the input neurons and recurrent network during an episode with many-to-many connections. Smoothed firing rate (binned in 1/7 s and smoothed with a causal exponential kernel, τ = 1/7 s, over 3 bins) demonstrates an example of recurrent network activity (right) leading the input neuron activity (left) throughout the progression of an episode near the **(A)** beginning, **(B)** middle, and **(C)** end. Recurrent neurons are matched to each input neuron and are redundantly represented for each input neuron they receive synapses from leading to the appearance of more background noise. Color scale ranges from dark blue to dark red representing a 0–20 Hz firing rate, respectively.

Activity in the network is initially the direct result of activated input neurons whether from noise or as part of the episode. As the network adapts to the input episodes, the background noise is suppressed and missing components of the repeated episode are activated along with the neurons that are soon to be activated. Background noise is suppressed due to recurrent inhibition and the down regulation of input scaling so as to counter the up regulation of recurrent connections between members of ensembles. The co-activation of neurons over several presentations allow for the learning of an episode on the basis of partial activity of member neurons in the ensemble when the partial pattern is reactivated as shown in Figure 4.

**Figure 5:**
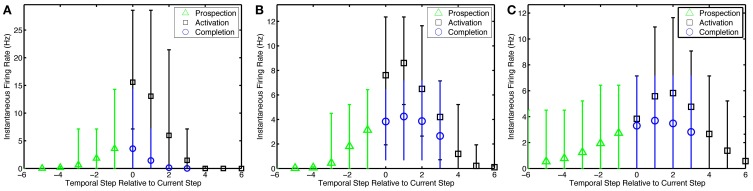
**Pattern prospection and completion in the simple model**. Instantaneous spike firing rates (in 1/7 s window) relative to the current step in the episode for the **(A)** simple model, **(B)** preplay model, and **(C)** preplay model with slower progressing stimulation with increased overlap. Negative lags are yet to be stimulated. Green triangles, black squares, and blue circles represent the average firing rate of recurrent neurons that are soon to be, currently being, and are not but would normally be stimulated via input neurons. Thus, they highlight prospective, active, and pattern completing neural activity, respectively. Bars indicate the 5–95% percentiles of firing rates for each marker.

With 1-to-1 input structure, the simple network neurons are excitable relative to their history of activity as expected. With the many-to-many input structure, the alternate network neurons that are active in fewer ensembles are excited stronger than other neurons in an ensemble. Neurons become active prospectively firing prior to the presentation of input that occurs in subsequent temporal steps. Neurons, also, complete missing portions of the patterns presented. The amplitude of the pattern completion and prospective activity is less than that of neurons directly activated by episodic input (see Figure 5). The prospective and pattern completion firing rates formed a smooth curve while the directly stimulated activity was far greater. This leads to the notion that the prospective firing and pattern completion are related by a common mechanism in this model, and that the prospective activity is pattern completion of the representation forward in time. However, see Noise Effects for differences in the learning of these phenomena.

### Synaptic weight changes

To examine learning at the synapses, LLEs of the excitatory and inhibitory weight spaces were performed over the course of learning. Briefly, LLEs map high dimensional spaces into neighborhood preserving low dimensional spaces (Roweis and Saul, 2000). The globally mapped nature of the low dimensional embedding makes them appropriate for visualizing data and relative trajectories in high dimensional spaces. Excitatory and inhibitory dimensionality reductions were performed separately due to the different time constants and learning rules involved. L_2_ distance in the weight space served as a poor indicator of learning (data not shown) and asymptotically demonstrated strong relation to the simulation time difference between weight vectors during learning.

**Figure 6:**
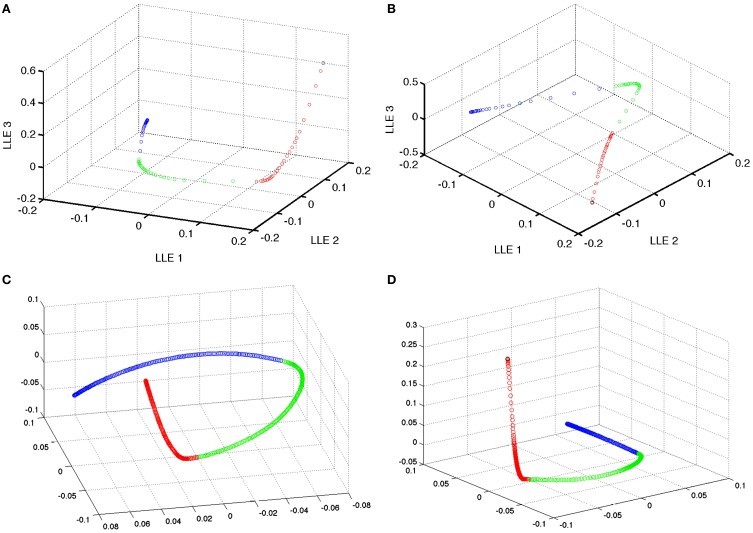
**Dimensionality reduction of the model weight space**. Reduction of the simple model 230,400 weight space [of **(A)** excitatory and **(B)** inhibitory recurrent synapses] to 3 dimensional space using local linear embedding. Black points are the initial state of the weights. Red points indicate the learning of the first group of 4 patterns. While green and blue represent the learning of the second and third patterns, respectively. Note that the weight changes are nearly orthogonal in the reduced dimensional space. There is some effect in the neighborhood finding process that blends between neighboring points (and thus episodes) at the tail of each segment of the episodic learning except the last one. **(C,D)** Excitatory and inhibitory, respectively, weight space reduction for the preplay model.

**Figure 7:**
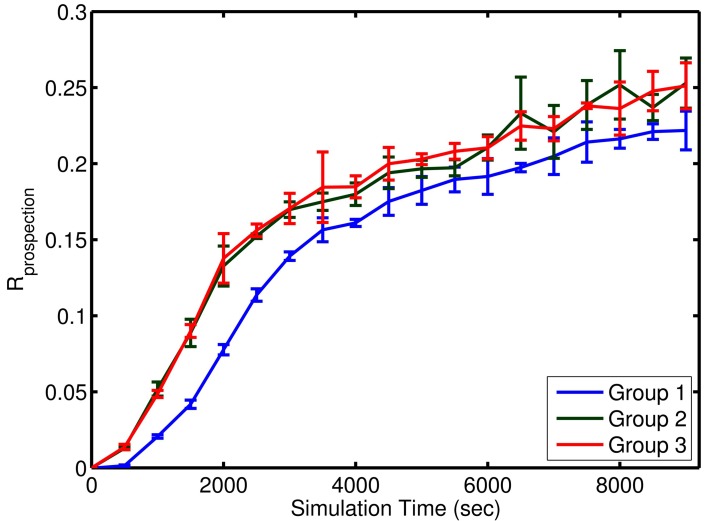
**Approximate learning curve of prospection in the simple model**. Each group of episodes is shown aligned to the first presentation of that group at time 0. Learning for Group 1 was slower due to the initial network adjustment in homeostatic values and the settling of synaptic weights at limit values. Learning occurred at the same rate for the two subsequent groups. Each learning curve represents the mean performance across four different simulation runs with four noise levels (*n* = 4) within a group. The error bars represent the standard deviation in *R*_prospection_ for that group at various episode durations.

Instead, the distance in a few dimensions of LLE space appeared to be a better indicator of changes in the network. For example, in a representative example, comparing three sequential sets of learning in the LLE space results in the expected non-linear adaptation and convergence of weights of recurrent network weights in the simple model. In this 3D LLE, these adaptations move along three nearly linear trajectories, each of which are nearly orthogonal (see Figure 6). This shows that given a global embedding into a low dimensional space, the weight vector trajectory moves from neighborhood to neighborhood in such a way that global changes are not discordant. During adaptation, the targeted correlated activity reduces inhibition, which in turn enables faster spiking and thus faster weight changes (see Figure 7). Weight adaptation rate peaks and slows as the pattern is learned and weights reach their maximum or minimum values. This coincides with a peak in the combined degree of prospectivity (i.e., look ahead time window) and pattern completion ability of the network (see Figure 5). Increasing the number of episodes (redundant allocation) that were being learned resulted in the trajectory of the learning in the first three LLE dimensions to appear less orthogonal (data not shown). This implies that learning of the groups of patterns does not interfere with each other when the subsequent uses of neurons are in completely new ensembles; however, pairwise reuse of neurons within the STDP windows violates this condition.

### Noise effects

**Figure 8:**
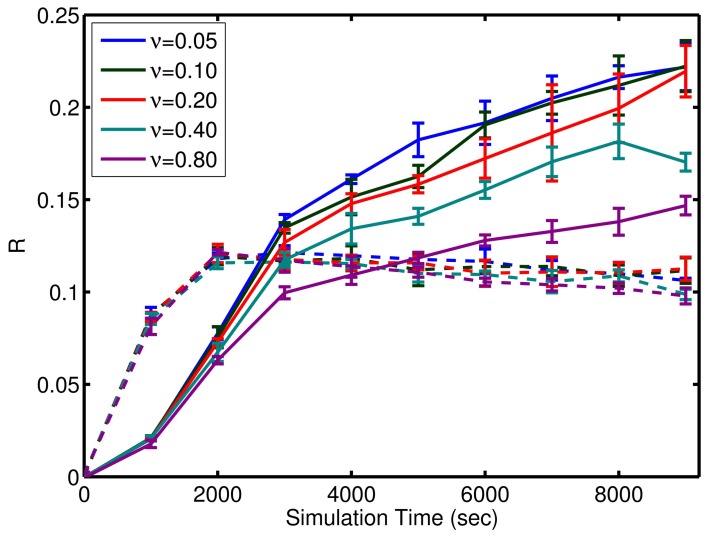
**Effect of noise on the learning**. Performance of the simple model through learning with incomplete patterns is shown here. Solid lines are the metric, *R*, for prospection. Dashed lines are the metric, *R*, for pattern completion. Note, the similar slope of the curves initially as compared to divergence in the final value relative to noise, ν. ν is expressed in terms of per neuron noise spikes in Hz. A significant amount of noise tolerance exists considering the input signals are an average of 6 spikes per presentation of each episode (which equates to a brief instantaneous firing rate of 10 Hz but averages out to 0.3 Hz over the course of the learning trial). The error bars represent the standard deviation in either *R*_propsection_ or *R*_completion_ for various episode durations. The results also show that the network is more robust to noise for pattern completion (solid lines) compared to prospective recall (dashed lines).

To define a metric to quantify network prospectivity and pattern completion, the firing rate of neurons for up to 2 temporal steps into the future of the episode or the neurons from the current step that were removed from the input are averaged and compared with the activity of the directly stimulated neurons.

(8) $$R=\frac{\mu_{\left(\text{prospection}|\text{completion}\right)}-\mu_{\text{background}}}{\mu_{\text{active}}-\mu_{\text{background}}}$$

where μ is the mean firing rate. Given that the inputs are not active for prospection or completion, this relative activity measure is expected to be less than 1 as homeostatic input scaling regulates neuron activity based on inputs as well as recurrent connections. Remarkably, the network is tolerant to large amounts of background noise for pattern completion but less so for prospective firing (see Figure 8). In contrast, the noise has little effect on the initial learning rate, but affects the final quality of learning (see Figure 8).

### Preplay and reverse replay

**Figure 9:**
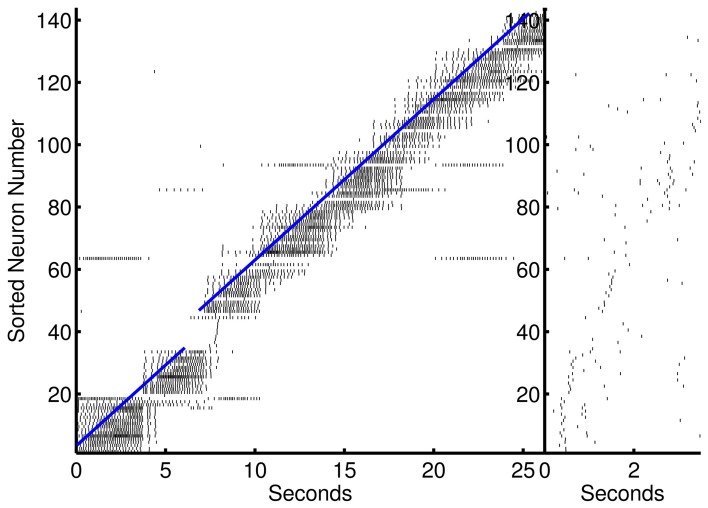
**Preplay of an episodic spike pattern**. Left, activation of the episode that was previously learned. Activity to the left of the blue line is prospective. Right, fast preplay of the episode to the right.

The preplay network began to demonstrate prospection sooner than other models (on the second presentation of an episode—however each step is presented 7× longer than the other models presented), and, in general, produced better and more stable pattern completion and prospective activity than the other models presented in this paper. Although more divergent from biological underpinnings, this model was able to demonstrate preplay and replay of activity in the form of self-sustaining sequences in forward or reverse, respectively. This is accomplished by reactivating the ensemble at the beginning (preplay) or end (replay) of the spatiotemporal sequence after learning has occurred. The episode is reactivated by short gamma frequency bursts of 1–4 spikes from each neuron in the initial or end segment of the episode (two temporal steps were used). This could be considered reminiscent of the upstream spiking activity due to the sensory input at the beginning or end of a linear track. The preplay of activity (see Figure 9) can be viewed as a full recall of a previous memory. The recall of previous memories based on partial patterns means that the results of various behaviors can be predicted given a similar sensory match to other experiences. The model also exhibits various successful durations of the preplay and replay of the entire episode that is qualitatively similar to the results to other recent research (Foster and Wilson, 2006; Diba and Buzsaki, 2007).

However, there are several limitations of this version of the model. This model aggressively suffered interference and learned more slowly the second and third sets of patterns. The sequence preplay speed did not occur on biologic timescales. The activity propagated at a much slower rate due to the high membrane capacitance resulting in long time constants and the slow inhibitory currents. The preplay propagation speed increased when using the fast stimulation protocol used for the other models due to depression of inhibitory weights. The capacitance and the localized slow inhibitory currents proved to be the reason for stability in this model; reducing the capacitance, as in the other models, results in less stability, yet, faster response times in the cascade or sequencing of activity. When forming symmetric excitatory connections between neurons (in the triplet STDP case, and needed for reverse replay), faster response times gives the network the ability to recruit a major portion of the network and this in turn leads to bursting activity in the network. A better method of achieving a balance between the stability-sustainability trade-off in the model could come from compartmental models and more robust and targeted inhibition.

The preplay model was only able to demonstrate replay when the triplet STDP rule was used in conjunction with a temporal blurring of the input patterns as opposed to the sharp probability peaks seen in Figure 2. A preplay model with traditional couplet STDP could learn, form prospective activity, and demonstrate preplay, however, reverse replay could never be achieved even when using shifted or noisy windows (Babadi and Abbott, 2010). Another interesting aspect of this model was the fleeting fragments of episodes were reignited (primarily forward and on the same time scale as preplay) when not driven by an episode pattern but still receiving random noise inputs (supplementary video available online).

## Discussion

We demonstrated recurrent SNNs capable of learning episodes operating with missing, noisy, and unbalanced data was demonstrated. This learning is demonstrated by the prospective firing of neurons attributed to subsequent stimuli and the completion of missing portions of the patterns. This prospective firing is excited by recurrent connections from neurons in the currently active ensemble. The active ensemble contains neurons that are components of temporally adjacent (both previous and subsequent) ensembles; however, the previous neurons were just active and are generally in a refractory state and moderately inhibited.

These recurrent spiking models provide a means to store patterns and recall or even predict them given a previously encoded pattern (Lisman and Redish, 2009). In the many-to-many input coding of the alternate model, the input to recurrent network mapping results in a randomized and more distributed encoding. This spreads activation through the recurrent neurons to preferentially recruit neurons that are not utilized or rarely utilized.

**Figure 10:**
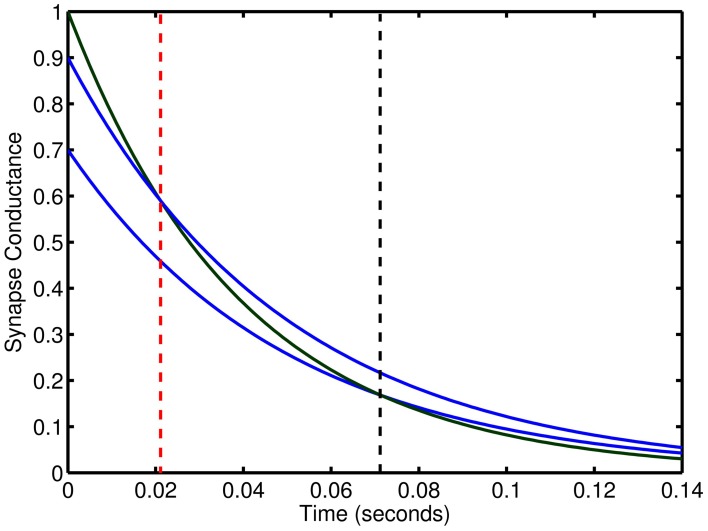
**Relative effect of synaptic connections with two time constants**. The temporal length of excitation can be controlled by changes in the weights of dual synapse with different time constants. Blue lines conductance trace examples at two weights for slow inhibitory time constant. Green line is an excitatory conductance trace. Vertical, dashed red and black lines show the time point at which inhibition begins to exceed excitation.

Our model maintains stability through strong high frequency inhibition to limit the network activity to be cyclic and by introducing the input pattern such that its driving force terminates at the peak of the low frequency cycle enabling recurrent activity to trail-off in the second half of the cycle. Therefore, the network is stable as long as network activity is always decaying which can be ensured by limiting the upside of excitatory weights, the downside of inhibitory weights, and using a time constant for inhibition that is slower than excitation. This balance enables the scaling of weights to modulate, on a neuron-to-neuron basis, the duration of time for which the net recurrent activity is excitatory before turning inhibitory. Setting the weight ranges so that the maximum duration is half of the low frequency cycle designates the spiking activity due to inputs to become inhibitory to the network and thus a stabilizing force (see Figure 10).

Other models exist which relate learning and sequences of spiking activity (O'Keefe and Recce, 1993; Rao and Sejnowski, 2001; Buonomano, 2005; Lisman et al., 2005). Another method proposed by Buonomano (2005), uses the scaling of the presynaptic component of the weight to learn a time delay and sequence a spiking pattern. However, those authors admit the difficulty with learning multiple different patterns. This work does not use the pattern correcting and forward lookup circuitry proposed in Lisman et al. (2005). However, this simple recurrent network is able to do both. As a result though, there is a tendency in this model to bring temporal associations forward and backward in the sequence learning—a separate pattern correction and completion network may solve this issue. Regardless, a spiking network that can perform free recall of multiple episodes has yet to be demonstrated in a simulated network that first learned the multiple episodes concurrently.

### Link to biology

Recurrent neural networks have been found throughout the brain (Rao and Sejnowski, 2001; Kobayashi and Poo, 2004; Buzsaki, 2006). Specifically, our network employs neural architecture design that can produce hippocampal-like behaviors including reactivation, preplay and replay (Pavlides and Winson, 1989; Wilson and McNaughton, 1994; Louie and Wilson, 2001; Andersen et al., 2006; Rasch and Born, 2007; O'Neill et al., 2008; Dragoi and Tonegawa, 2010; Gupta et al., 2010; Buhry et al., 2011). This enables the network to automatically load balance densely coded downstream networks through homeostatic regulation and sparse upstream coding. Furthermore, the low and high frequencies used here are similar to the theta and gamma rhythms found in the hippocampus (Buzsaki et al., 1992; O'Keefe and Recce, 1993; Skaggs et al., 1996; Penttonen et al., 1998; Bragin et al., 1999; De Almeida et al., 2007; Lenck-Santini and Holmes, 2008; Pastalkova et al., 2008; Zhang et al., 2011; Penley et al., 2012).

However, this network is appreciably simpler than the one found in the hippocampus, including the neural dynamics, learning rules, and homeostasis. The 80:20 ratio for excitatory to inhibitory connections is lower than that found in the CA3 region of the hippocampus but similar to cortical areas (Buzsaki, 2006). Moreover, we believe that the memory capacity of this network to be markedly smaller than of a more complex network where feedback inhibition is present in the form of multiple independent neurons and target specific regions of the principal cell's dendrites, soma, and axon. Also, the mammalian hippocampus is thought to encode through the sparse representation of the dendate gyrus (DG) and recall using a direct entorhinal cortex to CA3 pathway that contain several smaller connections as opposed to the DG-CA3 pathway (Treves and Rolls, 1992; Nolan et al., 2011) which was adopted here.

## Future work

Stability in this model is fragile with large weight changes, meaning that increased excitation or increased recruitment can easily lead to cascades of activity that result in bursts like many recurrent networks (Wagenaar et al., 2006). Once the network bursts, the network continues to burst due to both the positive feedback effect of triplet STDP and the negative feedback effect of inhibitory STDP during bursting. Separating late and early phase LTP (Adams and Dudek, 2005) may prevent infrequent occurrence of bursts from having these catastrophic ramifications. However, this may not be the case since an analogous development occurs *in vivo*; for example, tetanic hippocampal stimuli evolve into epilepsy (Sanchez et al., 2006). Alternatively, the use of reward-based learning methods may increase the speed and the specificity with which learning occurs by identifying new patterns and promoting learning via neuromodulation (Izhikevich, 2007b; O'Brien and Srinivasa, 2013).

In the preplay iteration of this model, there is evidence that this rudimentary kind of network supports replay and preplay at compressed timescales (Diba and Buzsaki, 2007). Due to the use of the triplet based rule, symmetric excitatory connections are formed between neurons allowing for both the forward or reverse propagation of activity. However, the triplet rule does not promote the robust symmetric connections found in the hippocampus with short bursts of post-then-pre-synaptic spikes. We believe that the use of a more phenomenological rule (Markram et al., 2012) would promote further symmetry in learning and robust replay on par with preplay without requiring as much blurring and overlap of spike trains. Further investigation of preplay is necessary to determine if the compression factor seen *in vivo* (Euston et al., 2007) occurs as a natural result of propagating neural activity without theta frequency resets.

### Conflict of interest statement

The authors declare that the research was conducted in the absence of any commercial or financial relationships that could be construed as a potential conflict of interest.
